# Vitreous lavage fluid and bronchoalveolar lavage fluid have equal diagnostic value in sarcoidosis

**DOI:** 10.1097/MD.0000000000005531

**Published:** 2016-12-09

**Authors:** Kazuichi Maruyama, Tohru Inaba, Tsutomu Tamada, Toru Nakazawa

**Affiliations:** a Department of Ophthalmology and Visual Science, Tohoku University Graduate School of Medicine, Sendai; b Infection Control and Laboratory Medicine, Kyoto Prefectural University of Medicine, Kyoto; c Department of Respiratory Medicine, Tohoku University Graduate School of Medicine, Sendai, Japan.

**Keywords:** bronchoalveolar lavage fluid, flow cytometry, sarcoidosis, T-lymphocyte, vitreous lavage fluid

## Abstract

Here, we elucidate the immunological features of both bronchoalveolar lavage fluid (BALF) and vitreous lavage fluid (VLF) samples from patients with histopathologically verified sarcoidosis. In addition, we assess the safety of vitrectomy in sarcoidosis patients by investigating the occurrence of complications and the recovery of visual acuity.

Twenty-two eyes of 22 patients with tissue-proven sarcoidosis were enrolled in this study. BALF and VLF samples were obtained and compared in each patient, and the clinical course (including visual acuity) was followed. The presence of sarcoidosis was assessed with a flow cytometric analysis of T-lymphocytes in the BALF and VLF samples.

Our results indicated that the CD4 T-cell population and the CD4/CD8 ratio were significantly higher in the VLF T-lymphocytes than the BALF T-lymphocytes. On the other hand, the CD8+ T-cell population was significantly lower in the VLF T-lymphocytes.

Therefore, our findings suggest that VLF samples have a high diagnostic value (equal to that of BALF samples) for sarcoidosis. Moreover, we found that the sample collection did not affect visual acuity and that there were no adverse events after surgery. A flow cytometric analysis of a VLF sample may therefore be a useful adjunct in the diagnosis of sarcoidosis.

## 1 Introduction

Sarcoidosis is an idiopathic inflammatory disorder characterized by noncaseating epithelioid cell granulomas in multiple tissues and organs, including the eye.^[1,2]^ The disease most commonly affects the lung, but other tissues (such as skin, the heart, and central nervous system, in addition to the eye) may also be affected.^[3]^ The most common signs of sarcoidosis in clinical examinations are therefore usually related to the respiratory system. Abnormal chest radiography findings are especially common, including findings of bilateral hilar lymphadenopathy and/or pulmonary infiltration. Previous reports have shown that fiber optic bronchoscopy can be used to obtain transbronchial lung biopsies for the histological diagnosis of sarcoidosis.^[4,5]^ The basic diagnostic criteria for sarcoidosis include the histopathological verification of noncaseating epithelioid cell granulomas and the exclusion of other diseases with granuloma formation reactions (such as tuberculosis).^[3]^

Immunological findings from clinical examinations are also important for diagnosing sarcoidosis.^[3]^ It has been reported that an increased CD4+ helper T-cell lymphocyte subset or a CD4/CD8 ratio greater than 3.5 in bronchoalveolar lavage fluid (BALF) is helpful in the diagnosis of sarcoidosis.^[6]^ BALF lymphocytosis (i.e., a CD4/CD8 ratio >3.5) and other laboratory features highly consistent with sarcoidosis, such as high serum levels of angiotensin-converting enzyme (ACE), can be reliable indicators when diagnosing sarcoidosis in cases where histology is not available.^[7,8]^ Moreover, our previous results indicated that the T-lymphocyte population in the vitreous lavage fluid (VLF) also has a high diagnostic value for diagnosing ocular sarcoidosis.^[9]^

It has been reported that 30% to 60% of sarcoidosis patients develop ocular lesions^[10–13]^ and that bilateral anterior or posterior uveitis are also common. The clinical appearance of sarcoidosis uveitis is characterized by iris nodules, mutton-fat keratic precipitates, and tent-shaped peripheral anterior synechiae in the anterior segment. In the posterior segment, phlebitis and vitreitis with snowball-like vitreous opacities are common findings in sarcoidosis uveitis.^[14]^ Moreover, persistent ocular chronic inflammation can cause epiretinal membrane and cystoid macular edema, leading to severe visual impairment.^[15,16]^ Internationally acknowledged criteria have been established for the diagnosis of sarcoidosis with ocular involvement.^[17]^ Although it is generally not difficult to diagnose ocular sarcoidosis with typical clinical findings, a considerable number of sarcoidosis cases present with nonspecific vitreous opacities. In these cases, differential diagnosis can be difficult.^[18]^ Moreover, analysis of VLF samples from sarcoidosis patients is a safe and useful way of diagnosing sarcoidosis uveitis with vitreal opacities. Flow cytometric analysis has also been reported to be useful in diagnosing uveitis,^[19]^ especially in sarcoidosis.^[9]^ Therefore, in the present study, we attempted to determine whether the diagnostic value of vitreous-infiltrating T-lymphocytes for sarcoidosis could be considered to be as high as that of BALF lymphocytosis.

## 2 Materials and methods

Patients who visited the uveitis clinic at Tohoku University Hospital or Kyoto Prefectural University from 2008 to 2015 and met the inclusion criteria were invited to participate in this study. This prospective study was approved by the Institutional Review Board of Tohoku University Graduate School of Medicine and Kyoto Prefectural University. All experimental procedures were conducted in accordance with the tenets set forth in the Declaration of Helsinki. The present study was registered in the University Hospital Medical Information Network Clinical Trial Registry. The purpose of the research and the experimental protocols were explained in detail to all patients, and their informed consent was obtained before participation in this study.

### 2.1 Participants

A consecutive series of 22 eyes with uveitis of 22 patients were enrolled; all eyes had received a definitive, tissue-proven, diagnosis of sarcoidosis, confirmed according to international diagnostic criteria.^[17]^ The patients included cases of uveitis with visual disturbance due to epiretinal membrane. All patients agreed to undergo pars plana vitrectomy (complete vitrectomy) and take part in the study. Patients were excluded if they had any prior history of intraocular surgery, especially vitreous surgery.

The patients in this study who were diagnosed histologically had positive biopsy results with suggestive intraocular signs and 2 positive investigational tests (a negative tuberculin skin test, elevated ACE, a positive sign on a gallium-67 citrate centigram, a high CD4/CD8 ratio in BALF samples, and high serum or urine calcium concentration). Most patients underwent BALF examinations before the ocular surgery. In all cases except 2, the patients underwent BALF examinations before receiving any steroid treatment.

### 2.2 Vitreous sample collection procedures

**Figure 1:**
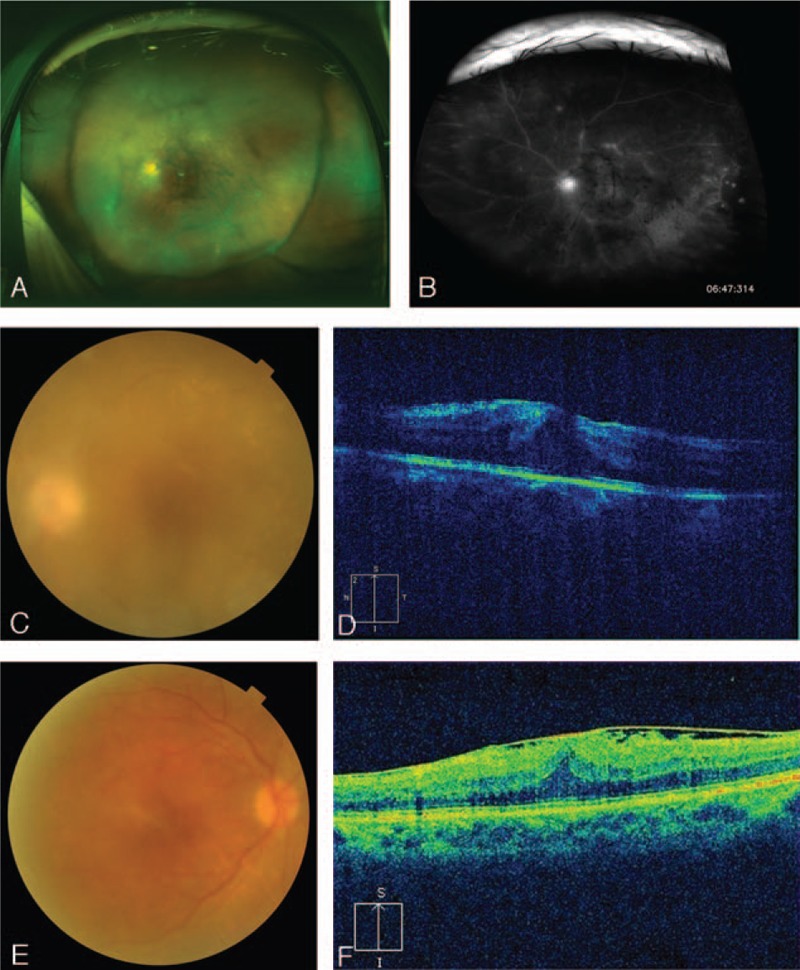
Representative clinical appearance at posterior segment of a sarcoidosis patient. The appearance of the posterior segment included a vitreous opacity, abundant exudate in the retina (A) and tubercular venous vasculitis (B). Severe vitreous opacity (C) and macular edema (D) were seen in a sarcoidosis patient who was scheduled for vitrectomy (D). The patient with preretinal membrane in the macular area was scheduled for vitrectomy (E and F).

At the start of a conventional 25-ga pars plana vitrectomy, a vitreous specimen was obtained from each patient. Typical presentations included severe vitreous opacity with retinal vasculitis (Fig. 1A and B), macular edema (Fig. 1C and D), and epiretinal membrane (Fig. 1E and F). The procedure used a Constellation (Alcon Laboratories, Inc., Fort Worth, TX) vitrectomy system. Our experimental protocol was based on a method we have previously reported.^[9]^ A 3-way stopcock was attached at the point the suction tube line connects to the cutter probe, and a 10-mL syringe was then attached to the free end of the 3-way stopcock. Dry vitrectomy was then performed without balanced salt solution (BSS; Alcon) perfusion. A cutting rate of 500 cpm was used to avoid damaging the cells infiltrating the vitreous. After a 1.5- to 3-mL sample of pure vitreous fluid was collected, the normal cutting rate vitrectomy (5000 rpm) continued with BSS perfusion. All of the obtained samples were promptly stored at 4 °C and brought to the clinical laboratory of the University hospital for cell analysis (including flow cytometry). All samples were immediately analyzed using the method described below.

### 2.3 Bronchoalveolar lavage fluid examination

At the beginning of the procedure, first the upper and then the lower airways were anesthetized. After confirming the effect of the anesthesia, bronchoalveolar lavage was performed by instilling and immediately retrieving 3 50-mL aliquots of warmed normal saline. Lavage was performed in only 1 site, either the right middle lobe or lingula. The quantity of recovered fluid was generally about 50% of the quantity of the initial injected fluid. This method follows previous reports.^[20]^

### 2.4 Flow cytometry analysis of the vitreous samples

Using a method we have previously reported,^[9]^ the VLF and BALF samples were filtered with a 70-μm cell strainer (BD Falcon Cell Strainer; BD Biosciences, Bedford, MA). The samples were then washed and resuspended with phosphate-buffered saline (Nissui, Tokyo, Japan) containing 2% bovine serum albumin (Nacalai Tesque, Kyoto, Japan) and 0.1% sodium azide (Nacalai Tesque) at a final volume of 0.5 mL. For the flow cytometric analysis, 0.1 mL of each sample was incubated with one of the following monoclonal antibody mixtures: fluorescein isothiocyanate (FITC)-conjugated mouse Immunoglobulin (Ig)G1 (Dako Denmark AS, Glostrup, Denmark)/phycoerythrin (PE)-conjugated mouse IgG1 (Dako)/PE-Cy-Chrome5-conjugated anti-CD45 (clone T/29/33; Dako), anti-CD3-FITC (UCHT1; Dako)/anti-CD19-PE (HD37; Dako)/anti-CD45-Cy5 (T29/33; Dako), or anti-CD4-FITC (T4; Beckman Coulter, Miami, FL)/anti-CD8-PE (T8; Beckman-Coulter)/anti-CD3-Cy5 (UCHT1; Dako). After a 15-minute incubation period at room temperature in complete darkness, the samples were again washed and resuspended at a final volume of 0.5 mL. Lymphocyte subsets of the VLF and BALF samples were examined with an Epics XL-MCL flow cytometer (Beckman Coulter). CD45^+^ cells were initially counted on CD45 versus side-scatter plots combined with forward scatter and side scatter. If the counts determined that more than 100, CD45^+^ cells were contained in 1 test tube, further analysis was then performed to determine the T-cell (CD3^+^, CD45^+^)/B-cell (CD19^+^ and CD45^+^) ratio, as well as the CD3^+^, CD4^+^ T-cell/CD3^+^, CD8^+^ T-cell ratio. The remaining portion (0.2 mL) of the resuspended VLF and BALF samples were prepared for cytology with a cytocentrifuge (Cytospin 4; Thermo Fisher Scientific, Inc., Waltham, MA).

Flow cytometry was also used to study the BALF lymphocyte population. The specimens were washed and then resuspended at a final volume of 0.5 mL/tube.

### 2.5 Clinical course assessment after vitrectomy using visual acuity change

All patients underwent vitreous surgery; visual acuity was analyzed both before and after surgery. All patients were followed at the outpatient clinic of our facility for at least 1 month postoperatively. Visual acuity was measured at every visit as the logarithm of the minimum angle of resolution (log MAR). Previtrectomy and postvitrectomy measurements were compared. Postoperative visual acuity was recorded as the last measurement obtained in the outpatient clinic (the average observation period was 20.5 ± 14.8 months). The patients were also monitored for adverse events after vitreous surgery.

### 2.6 Statistical analysis

Between-group statistical comparisons of the CD4^+^ and CD8^+^ T-cell population and of the CD4/CD8 ratio were carried out with a nonparametric analysis based on the Mann–Whitney test (significant differences were defined as *P* < 0.05). Analyses were performed with Prism software, version 5.0.1 (GraphPad Software, San Diego, CA). Visual acuity before and after vitrectomy was analyzed using the paired *t* test. These analyses were performed with Prism software, version 5.0.1 (GraphPad Software).

## 3 Results

### 3.1 Characteristics of patients

**Figure 2:**
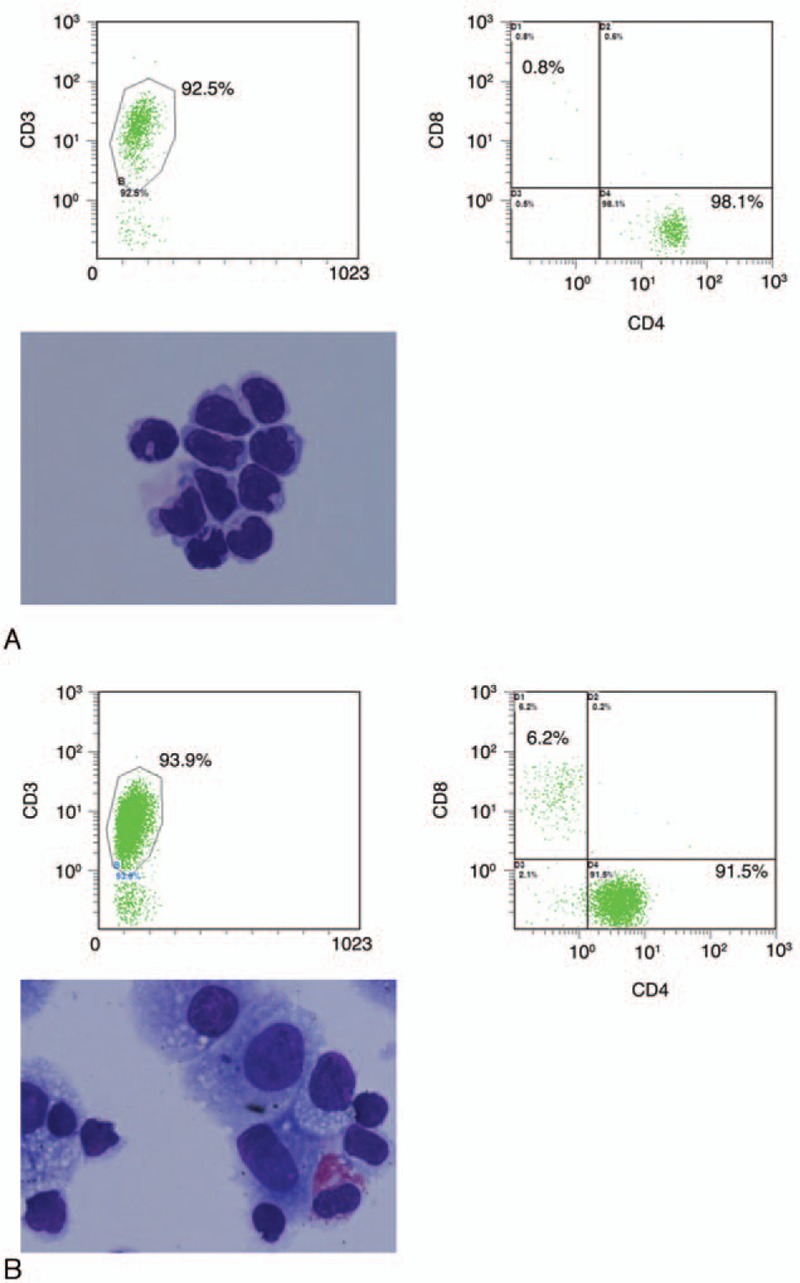
Representative flow cytometry and cytology data. Representative flow cytometry and cytology data from analysis of vitreous lavage fluid (A) and bronchoalveolar lavage fluid (B).

This study included 22 patients (18 female and 4 male). The mean age was 67.6 ± 5.4 years. We analyzed flow cytometry data from the BALF and VLF samples in order to search for differences in the cell populations of these 2 fluids (Table 1). In 15 cases, BALF was examined in the patients before VLF. In 7 cases, VLF was examined before BALF because the patients had severe vitreous opacities and it was necessary to gather data for a differential diagnosis of primary intraocular lymphoma. All patients underwent vitrectomy due to recurrent macular edema, epiretinal membrane, and severe vitreous opacities. Representative data from the BALF and VLF cell analyses are presented in Fig. 2. Before the samples were analyzed, we confirmed that they contained a sufficient number of cells. Most importantly, we confirmed that the VLF samples contained more than 100 CD45^+^ cells. It is commonly known that the vitreous cavity contains only 4 to 5 mL of VLF in adults, meaning that the volume of the VLF samples was necessarily less than the BALF samples. This risked affecting the validity of the comparisons, prompting us to use a perfusion system in the vitreous cavity to collect a sufficient number of cells during vitrectomy. Both the VLF (Fig. 2A) and BALF (Fig. 2B) samples contained mononuclear cells expressing high levels of CD3. This CD3^+^ population was used to check CD4 and CD8 expression.

**Table 1:**
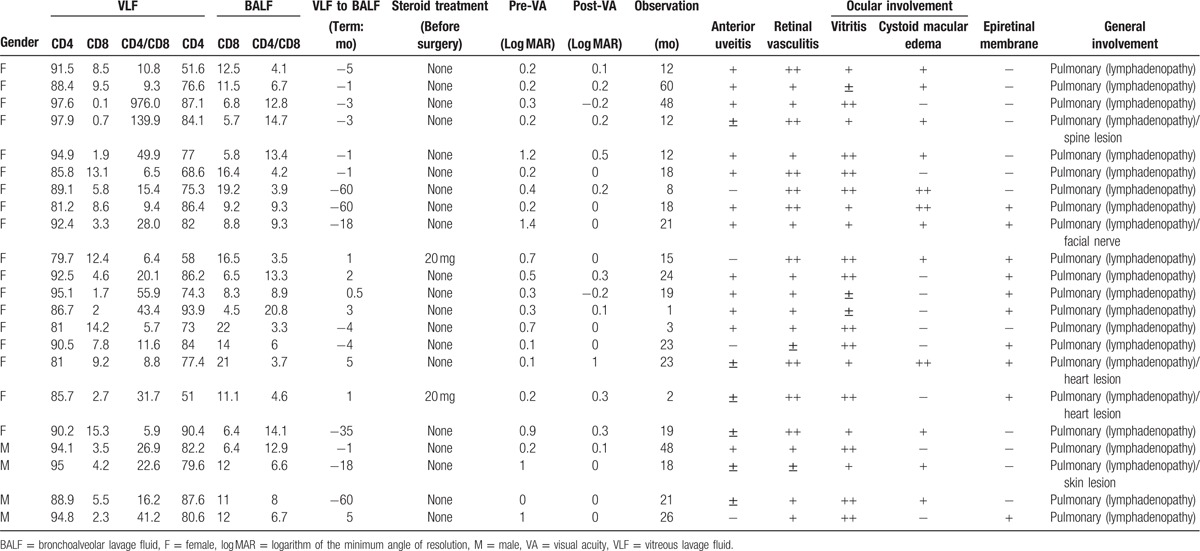
. Patient's whole data.

### 3.2 Comparison of flow cytometric analyses in vitreous fluid and bronchoalveolar fluid samples

**Figure 3:**
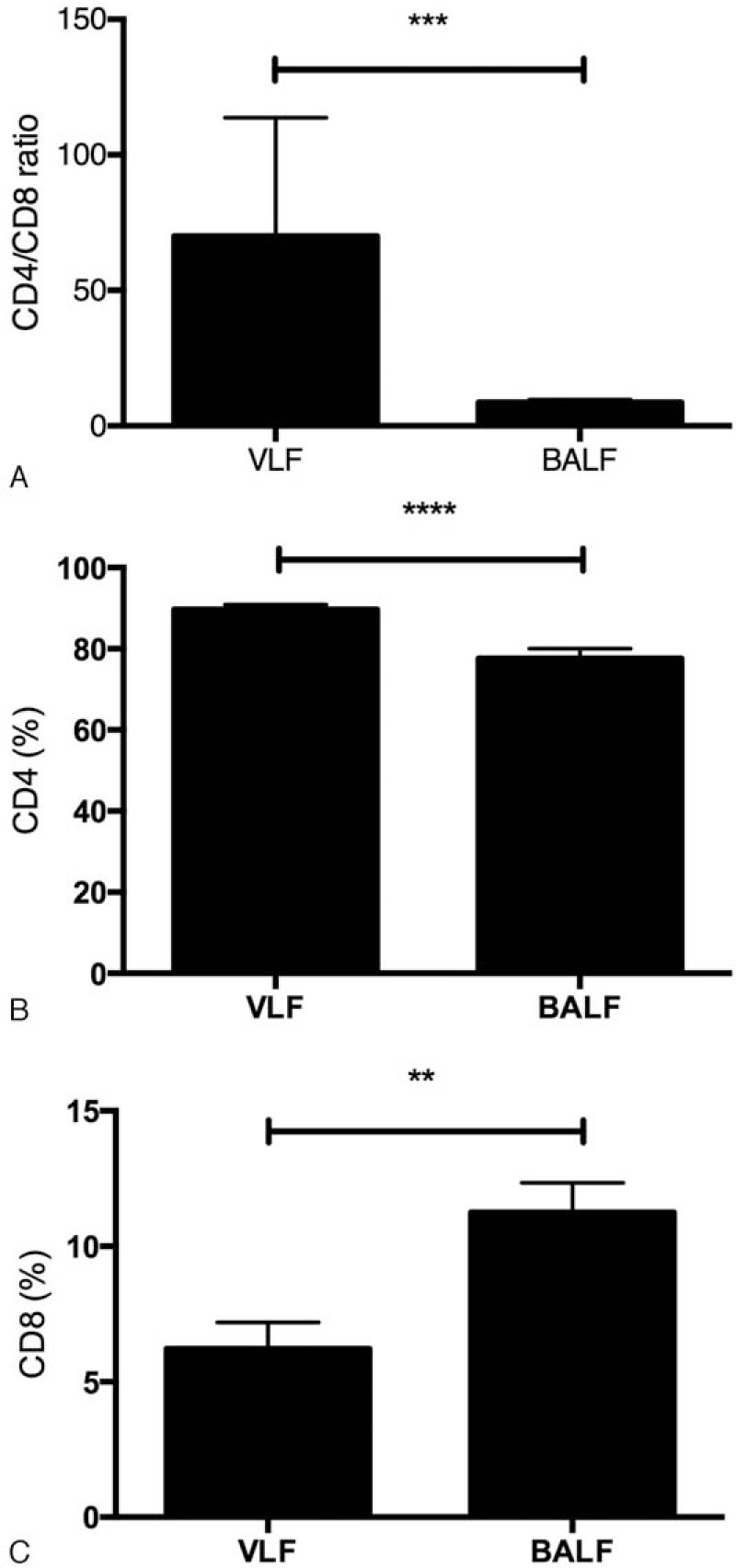
Quantification of T-lymphocyte subsets. Quantification of T-lymphocyte subsets in vitreous lavage fluid and bronchoalveolar lavage fluid. CD4/CD8 ratio (A), CD4 (B), and CD8 (C). ^∗^*P* = 0.0018, ^∗∗^*P* = 0.0006, ^∗∗∗^*P* < 0.0001.

Flow cytometric data for the CD4 lymphocytes, CD8 lymphocytes, and CD4/CD8 ratio are summarized in Table 1. In the VLF samples, the mean CD4/CD8 ratio was 70.1 ± 43.6. In the BALF samples, the mean CD4/CD8 ratio was 8.7 ± 4.7. The CD4/CD8 ratio of the lymphocytes obtained from the VLF samples was significantly higher (*P* = 0.0006) than the ratio of the lymphocytes in the BALF samples (Fig. 3A). Flow cytometric data for the CD4 lymphocytes are summarized in Fig. 3B. In the VLF samples, the mean CD4 percentage was 89.7 ± 5.5. In the BALF samples, the mean CD4 percentage was 77.6 ± 11.5. The CD4 percentage of the lymphocytes in the vitreous samples was significantly higher (*P* < 0.0001) than the BALF samples.

Flow cytometric data for the CD8 lymphocytes are summarized in Fig. 3C. In the VLF samples, the mean CD8 percentage was 6.2 ± 4.6. In the BALF samples, the mean CD8 percentage was 11.3 ± 5.1. The CD8 percentage of the lymphocytes obtained from the vitreous samples was significantly lower (*P* = 0.0018) than the BALF samples.

### 3.3 Clinical course of the patients

**Figure 4:**
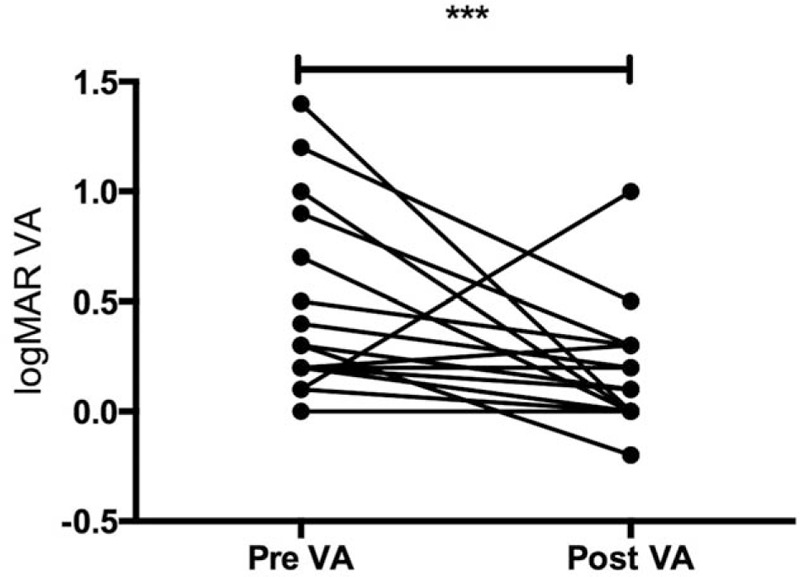
Quantification of visual acuity. Comparison of visual acuity (VA) in logarithm of the minimum angle of resolution units, preoperatively (pre-VA) and postoperatively (post-VA), in the sarcoidosis patients. ^∗∗∗^*P* < 0.003.

In the present study, the average visual acuity of the patients, measured in log MAR units, was higher after the operation (0.1 ± 0.3) than before (0.5 ± 0.4, *P* < 0.003) (Fig. 4). Moreover, almost all patients had better visual acuity after vitrectomy. However, 1 patient lost visual acuity after vitrectomy. This patient had severe retinal damage due to severe macular edema, retinal vessel occlusion, and retinal hemorrhage. Therefore, with 1 exception, the clinical course was favorable in almost all patients involved in this study, with no severe adverse events resulting from any of the surgical procedures, including the collection of the vitreous sample.

## 4 Discussion

This is the first evaluation of differences in the T-cell populations of BALF and VLF samples, taken from the same patient, in a study population with histopathologically confirmed sarcoidosis. In most of the patients included here, BALF was examined before VLF, and the examinations were performed before the patients received any steroid treatment. The major findings of this study were that the CD4/CD8 ratio and CD4 lymphocytes were elevated in VLF samples of patients with sarcoidosis. Moreover, in contrast to CD4, the percentage of CD8 lymphocytes was lower in the sarcoidosis VLF samples, to a similar degree as the elevation in BALF samples. Therefore, VLF can be considered a powerful tool for diagnosing sarcoidosis.

Previously, we reported that a diagnosis of ocular sarcoidosis based on a CD4/CD8 ratio among vitreous-infiltrating lymphocytes greater than 3.5 had a sensitivity of 100% and a specificity of 96.3%.^[9]^ Both of these values are remarkably higher than those for diagnoses based on BALF examinations.^[9]^ Indeed, our present results indicated that the CD4/CD8 ratio in VLF was higher than in BALF. We speculate that this phenomenon arises from specific features of the ocular tissues, especially the intraocular tissue. In particular, intraocular tissue is isolated from the innate immune system, in contrast to the lungs, which are constantly exposed to external environmental stresses. Both the presence of fewer spontaneous immune cells and lowered activation of the immune cells in ocular tissue may explain this lessened immune response. The previous results indicated that nonsarcoidosis diseases, such as infectious endophthalmitis, had a higher CD8 lymphocyte population than sarcoidosis. The high CD4 we observed in VLF therefore means that it likely reflects the pathological features of sarcoidosis more directly than BALF. We also speculate that the higher CD4/CD8 ratio in VLF than in BALF may be due to differing numbers of cells. Indeed, it is easier to collect a high number of cells in BALF than in VLF due to the ability to collect a higher sample volume. When staining cells with antibodies for flow cytometry, samples with a low number of cells will have more conjugation than those with a high number of cells. The antibody conjugation may therefore have become saturated in the VLF samples, contributing to a positive shift in CD4 in the VLF samples compared to the BALF samples.

According to the American Thoracic Society, bronchoalveolar lavage causes no complications or adverse events in up to 95% of cases. However, complications and adverse events such as cough, transient fever, transient chills or myalgia, transient infiltration, bronchospasm, transient reductions of lung function, and transient decreases in baseline partial pressure of O_2_ in arterial blood can arise, although most of these resolve quickly. Therefore, bronchoalveolar lavage is recognized as one of the best tools for collecting samples for use in diagnosing sarcoidosis. Previously, it was thought that collection of vitreous samples from uveitis patients should only be done with caution, due to the risk of complications, such as severe inflammation caused by the surgical procedure. However, compared with earlier vitrectomy procedures that used 20-ga instruments, current 25- or 27-ga vitrectomy is quick, has a short visual recovery time, and has improved patient comfort.^[21]^ Moreover, in this study, the final stage of the operation included the spraying of triamcinolone acetonide (MaQaid; Wakamoto Pharmaceutical Co., Tokyo, Japan) into the vitreous cavity, after which the cavity was washed and the redundant triamcinolone removed. Finally, a sub-Tenon injection of 20 mg of triamcinolone was performed to prevent complications. Vitrectomy has thus become a worthwhile and safe diagnostic tool for uveitis. Indeed, our present results indicated that most of the patients who underwent vitrectomy had improved visual acuity and no severe complications, with only a single patient experiencing a loss of visual acuity due to continuous cystoid macular edema before surgery. Therefore, the clinical course was favorable in most patients involved in this study, with no adverse events resulting from any of the surgical procedures, including the collection of the vitreous samples. Therefore, modern 25-ga vitrectomy, such as that used in the present study, can be considered a safe way to perform sample collection in uveitis patients, especially in sarcoidosis patients.

Previously, it was reported that in Japanese patients, ocular sarcoidosis symptoms are the most frequently seen symptom at first presentation.^[22]^ At the present time, a diagnosis of sarcoidosis with ocular manifestation requires additional systemic features for confirmation. However, a systemic search reveals considerable numbers of suspected ocular sarcoidosis cases with negative results.^[23]^ Transbronchial lung biopsies and bronchoalveolar lavage are said to be relatively less-invasive procedures, yet there is ongoing disagreement as to whether they should be performed on patients without clinical or radiological pulmonary presentation. The present results show that an elevated CD4/CD8 ratio and CD4 lymphocyte population represent focal lymphocytosis characteristic of ocular sarcoidosis. This immunological resemblance between VLF and BALF should become an important future part of the diagnosis of sarcoidosis since it is not observed in other types of uveitis.^[9]^

It remains important to distinguish between pulmonary tuberculosis and sarcoidosis, because the CD4/CD8 ratio in the BALF of tuberculosis patient has also been reported to increase. Clinically, the symptoms of tuberculosis uveitis resemble those of sarcoidosis uveitis, including such features as retinal vasculitis and retinal exudate. In the present paper, we used a tuberculin test to screen for tuberculosis in all patients and obtained negative results. However, the results of a tuberculin test are not enough by themselves to rule out tuberculosis. Therefore, we also performed a PCR analysis for tuberculosis DNA in the vitreous samples. Moreover, in patients with relatively high positive results in the tuberculin test, we also performed a T-spot analysis. The Japanese setting of this study provided few opportunities to examine tuberculosis uveitis patients undergoing vitrectomy. Nevertheless, if possible, we would like to include such patients in the future research in order to report their CD4/CD8 ratio.

In conclusion, we believe that the testing methodology and immunological information reported here constitute an effective new way to diagnose sarcoidosis in cases with an active lesion localized in the eye and that in the future this method should enable earlier and less-invasive diagnosis of this ocular inflammatory disorder.

## Acknowledgments

We would like to thank Dr Wendy Chao for editing and critical reading of this manuscript. The authors thank Drs Hiroshi Kunikata, Kenji Nagata, Kazuhito Yoneda, and Kentaro Kojima for the helpful sample collections.
